# *TaaI*/Cdx-2 AA Variant of *VDR* Defines the Response to Phototherapy amongst Patients with Psoriasis

**DOI:** 10.3390/life11060567

**Published:** 2021-06-16

**Authors:** Aleksandra Lesiak, Karolina Wódz, Magdalena Ciążyńska, Małgorzata Skibinska, Michał Waszczykowski, Karol Ciążyński, Irmina Olejniczak-Staruch, Dorota Sobolewska-Sztychny, Joanna Narbutt

**Affiliations:** 1 Department of Dermatology, Paediatric Dermatology and Oncology, Medical University of Lodz, 91-347 Lodz, Poland; malgorzata.skibinska@umed.lodz.pl (M.S.); irmina.olejniczak-staruch@umed.lodz.pl (I.O.-S.); dorota.sobolewska-sztychny@umed.lodz.pl (D.S.-S.); joanna.narbutt@umed.lodz.pl (J.N.); 2 Department of Molecular Biology, VET-LAB Brudzew, 62-720 Brudzew, Poland; wodz.karolina@gmail.com; 3 Nicolaus Copernicus Multidisciplinary Centre for Oncology and Traumatology, Department of Proliferative Diseases, 93-513 Lodz, Poland; ciazynska.magdalena@gmail.com; 4 Department of Arthroscopy, Minimally Invasive Surgery and Sports Traumatology, Medical University of Lodz, 90-549 Lodz, Poland; mwaszczykowski@wp.pl; 5 Institute of Applied Computer Science, Lodz University of Technology, 90-924 Lodz, Poland; karol.ciazynski@gmail.com

**Keywords:** psoriasis, *VDR* gene, narrowband phototherapy

## Abstract

1,25-dihydroxyvitamin-D_3_ plays a central role in the immune system via binding to the vitamin D receptor. *VDR* polymorphisms have been associated with multiple autoimmune disorders, including psoriasis. Until now, five *VDR* polymorphisms, *FokI*, *ApaI*, *BsmI*, *TaqI* and *TaaI*/Cdx2, have been studied in psoriasis, with contradicting results. Therefore, this study aimed to evaluate the association of *VDR* polymorphisms with susceptibility to psoriasis, effectiveness of NB-UVB phototherapy and concentration of proinflammatory cytokines and vitamin D amongst the Polish population. *VDR* polymorphisms were analyzed by PCR-RFLP or real-time PCR. We found that the frequency of the *TaaI*/Cdx-2 GG genotype was significantly higher in psoriasis patients and was associated with regulation of IL-17 and IL-23 concentration. Moreover, *TaaI*/Cdx-2 AA might have a significant effect on the response to phototherapy amongst patients with psoriasis. Our results suggest that VDR is a susceptibility factor for psoriasis development. Moreover, *TaaI*/Cdx-2 variants have a significant effect on the response to phototherapy amongst patients with psoriasis and regulation of inflammatory response via decrease of IL-17 and IL-23 level after UVB phototherapy in the Polish population. Results of our study provide some evidence in support of the hypothesis that the vitamin D signaling pathway may be of relevance for pathogenesis and treatment of psoriasis.

## 1. Introduction

Psoriasis is a chronic autoimmune skin disease with a genetic background [1]. Its prevalence is estimated to be 2–4% worldwide [2,3]. A Polish epidemiological study by Borzecki et al. [4] in which 1,147,279 patients with psoriasis were analyzed showed that prevalence of the disease in Poland is about 2.99%. As the disease mainly affects professionally active people, it is a real socio-economic problem [5]. The presence of skin lesions is associated with a decrease in patients’ quality of life which leads to impairment in their professional, social and family life. In recent years, there has been a breakthrough in the understanding of the pathogenesis of the disease in terms of the participation of genetic, immunological [6] and environmental factors [7]. Plaque psoriasis is the most common clinical form of psoriasis vulgaris and accounts for approximately 85% of all cases. The assessment of disease activity based on the PASI (Psoriasis Area and Severity Index) index, BSA (Body Surface Area) and DLQI (Dermatology Life Quality Index) measurements is a key element in making therapeutic decisions. It is believed that the involvement of more than 10% of the skin surface by psoriatic lesions is an indication for the initiation of phototherapy or general treatment. However, there are cases in which patients experience a significant reduction in the quality of life despite the fact that the lesions occupy a smaller area, which should be taken into account in the selection of therapy [8]. PASI as well as BSA and DLQI are used to assess the severity of plaque psoriasis. PASI or BSA > 10 or DLQI > 10 define moderate to severe psoriasis. PASI, BSA and DLQI ≤ 10 relate to mild psoriasis. As psoriasis is a chronic disease, the majority of patients need continuous treatment. In recent years due to new molecular achievements, psoriatic biological treatment has been introduced against tumor necrosis factor (TNF), interleukin IL-12 and IL-23, IL-23 (IL-23p19) and IL-17. Despite the new therapeutic options, classic systemic therapies are still used, i.e., MTx (Methotrexate), CsA (cyclosporin A), oral retinoids and phototherapy [8].

UV light therapy is very important in the treatment of patients suffering from psoriasis. It is an effective, cheap method that has relatively few side effects. Narrowband UVB phototherapy, also known as TL-01 or NB-UVB (Narrowband UVB), was introduced in the 1980s and has since become one of the most common methods of light therapy [9]. The European guidelines on the basis of several other studies have determined that the use of NB-UVB irradiation twice a week causes remission within 20 weeks in 63–75% of patients. The recommendations of the AAD (American Academy of Dermatology) indicate that the initial improvement may occur within two weeks, with an average of 15–20 treatments to induce remission, and after one year this remission is maintained in 38% of patients [10]. A literature review for NB-UVB estimated PASI 75 at 62%, and when assessing the percentage of patients achieving remission (or ≥90% improvement), it was on average 68% [11].

The study comparing the use of high doses of NB-UVB (initial dose of 70% MED; minimal erythema dose), with subsequent increases by 40%) with the use of low doses of NB-UVB (initial dose of 35% MED and increase of subsequent doses by 20%) showed a similar the effectiveness of both methods, but for high doses of NB-UVB the desired effect was achieved with a smaller number of irradiations and lasted longer [12]. It has also been shown that NB-UVB is more effective when used three times a week compared to the twice weekly regimen [13,14,15].

Since the introduction of lamps with a narrow spectrum of radiation, many studies have confirmed the superiority of NB-UVB over BB-UVB (Broadband UVB). The use of narrowband UVB can led to better treatment outcomes and faster regression of the lesions [10]. The meta-analysis evaluating the effectiveness of NB-UVB and BB-UVB in the treatment of psoriasis clearly showed that the use of NB-UVB brings better results, especially in relation to plaque psoriasis [11].

As in most diseases with a complex etiology, the response to treatment is individual and often difficult to predict based on our own observations and literature data, and it is assumed that individual response to treatment is genetically determined. Therefore, determining precise phenotypes will allow for the selection of the best treatment option for patients with psoriasis in the future [11].

Vitamin D serves as regulator of the immune system. Its action depends on the vitamin D receptor (VDR), a member of the nuclear hormone receptor superfamily. The *VDR* gene is located on chromosome 12q12-q14. The most frequently occurring *VDR* polymorphisms are *FokI* (rs2228570), *BsmI* (rs1544410), *ApaI* (rs7975232) and *TaqI* (rs731236). Recently, Yamamoto et al. [16] described a functional binding site for the intestinal-specific transcription factor Cdx-2 in the 1a promoter region of the *VDR* gene. Subsequently, Arai et al. [17] described a G to A nucleotide substitution *TaaI*/Cdx-2 (rs11568820) at this Cdx-2 binding site, which was found to modulate the intestine-specific transcription of the *VDR* gene. In particular, the A-allele was found to more efficiently bind the Cdx-2 protein in vitro and showed increased transcription activity of the *VDR* promoter compared with the G allele [18]. VDR is responsible for cellular effects of vitamin D and the regulation of various intracellular signaling pathways which are involved in cell differentiation [19,20]. Genetic polymorphisms in *VDR* influence the level of vitamin D synthesis in the skin, liver and kidneys, as well as its metabolism and degradation. VDR is expressed in the intestine, thyroid and kidneys and has a vital role in calcium homeostasis. VDRs repress expression of 1alpha-hydroxylase (the proximal activator of 1,25(OH)2D_3_) and induce expression of the 1,25(OH)2D_3_ inactivating enzyme CYP24. VDR is expressed on keratinocytes and is a natural ligand for calcitriol which has the ability to inhibit proliferation and induces differentiation of human keratinocytes [21,22].

In some studies, associations between several gene polymorphisms and response to treatment have been assessed. It was revealed that in the case of HLA-C*06, the risk allele carriers have different responses to systemic treatment with methotrexate and biologics [23,24,25,26]. In patients who inherit at least one HLA-C*06 variant, there is a better response to methotrexate [24] as well as to ustekinumab, an inhibitor of IL-12/23 [25,26]. However, it was also shown that HLA-C*06 carriers have a poorer response to the treatment of TNF-alfa inhibitors when compared to the patients without this allele [25]. Bojko et al. [27] performed a study in which they assessed the role of IL12B, IL23A and IL23R genetic variants in susceptibility and response to treatment with NB-UVB, however they found no significant result. To the best of our knowledge, this is the first study to find genetic evidence that the *VDR* gene is associated with effectiveness of phototherapy. The aim of our paper is to perform an association study between *VDR* polymorphism, proinflammatory cytokine and vitamin D levels, and individual response to NB-UVB phototherapy commonly used in the treatment of psoriasis.

## 2. Materials and Methods

### 2.1. Subjects

The study group included 50 patients with psoriasis vulgaris (PsV) treated with NB-UVB therapy at the Department of Dermatology, Medical University of Lodz. The mean age was 42 years old (18–63 years old) and the mean duration of the disease was 20 years (from 6 months to 40 years). Fifty unrelated healthy controls (age and sex matched, Caucasian of Polish origin) were enrolled into the study. All individuals gave written informed consent before entering the study. The experimental plan was approved by the local ethics committee of the Medical University of Lodz and was conducted according to the principles of the Declaration of Helsinki. Before treatment, the intensity of psoriatic lesions was assessed in all patients by the PASI, BSA and DLQI. The mean: PASI index before phototherapy was 21.38; BSA was 33.46; DLQI was 16.2 [28,29]. The patients had II and III skin phototypes as assessed by the Fitzpatrick scale. The patients underwent irradiations with UVB NB (wavelength 311–312 nm) for 20 consecutive days with initial dose of 0.7 personal MED (minimal erythema dose). They were treated in a Dermalight-Medisun 2800 PC-AB cabin (Schulze and Böhm GmbH-Brühl, Germany) with TL100W/01 fluorescent lamps (Philips, Eindhoven, Netherlands). The mean initial dose was 0.2 J/cm^2^ and it was systematically increased either daily or every other day, depending on the individual patient’s reaction. The mean cumulative dose of UVB 311 radiation was 12.7 J/cm^2^. This regimen has been used at our department for several years and treatment results are satisfactory. The same doses were used also in other studies [30].

### 2.2. ELISA

Blood samples were taken amongst all subjects, once for DNA genotyping and twice to determine the concentration of TNF alpha (ELISA kits (Human HS TNF-α, R&D Systems, Minneapolis, MN, USA), IL-17 (Raybiotech INC. Norccross, GA, USA) and IL-23 (R&D System, Minneapolis Abingdon, MN, USA) with the use of ELISA according to standard manufacturer’s protocol. Moreover, in each subject the level of 25(OH)D and lipids, glucose and liver was assessed. These procedures were also repeated after the 20th irradiation.

### 2.3. Genotyping

DNA was extracted from whole blood using spin column-based DNA extraction kits, according to the manufacturer’s instructions (A&A Biotechnology, Gdańsk, Poland). The occurrence of the SNP: four *VDR* (rs2228570, rs7975232, rs1544410, rs731236) gene sequences were assessed in the patients with psoriasis and controls by a standard PCR-RFLP method with using DreamTaq Green PCR Master Mix (2X) (Thermo Scientific™, Waltham, MA, USA). PCR conditions were as follows: 1 cycle at 95 °C for 3 min for an initial denaturation, followed by 35 cycles of denaturation for 30 sec at 95 °C, primer annealing for 30 sec at 56 °C, primer extension for 1 min at 72 °C and a final extension for 7 min at 72 °C. The loci were recognized by FastDigest^®^ restriction enzymes (Thermo Scientific™, Waltham, MA, USA), used at 37 °C for 20 min, except for *TaqI* which was used at 65 °C for 20 min. RFLP products were electrophoresed for 45 min and visualized on 2% agarose gels stained with ethidium bromide. The size of the restriction endonuclease digested products was determined using a GeneRuler 50 bp DNA Ladder (Thermo Scientific™, Waltham, MA, USA). A TaqMan^®^ SNP Genotyping assay was used to detect the single nucleotide polymorphism rs11568820 (*TaaI*/Cdx-2—*VDR*, A/G). Real-Time PCRs were performed according to the manufacturer’s instructions in a 96-well format in a total reaction volume of 5 μL using 10 ng of genomic DNA and ABI Prism 7900HT (Thermo Scientific™, Waltham, MA, USA). Thermal conditions were as follows: initial denaturation at 95 °C for 10 min, 40 cycles were run at 95 °C for 15 s (denaturing) followed by 60 °C for 1 min (annealing/extension).

Details of the experimental conditions are shown in Table 1.

### 2.4. Statistical Analysis

Qualitative variables are presented as numbers with a corresponding percentage. The chi-square test with appropriate corrections applied depending on the size of the subgroups was used for the analysis. Continuous variables are presented as median with the values of the lower and upper quartiles (25–75 percentile). The normality of the distribution was verified by the Shapiro-Wilk W test. The differences between the groups were assessed using the Mann-Whitney and Kruskal-Wallis test or the χ^2^ test (variables with a distribution other than normal). Spearman’s rank correlation was used to determine the correlation of variables with a distribution other than normal. The differences for the statistic value *p* < 0.05 were considered statistically significant. The statistical analysis was performed using the Statistica 13.1 software package (StatSoft, Krakow, Poland). *p* value ≤ 0.05 was considered statistically significant.

## 3. Results

### 3.1. Influence of UVB Treatment on Selected Clinical Features

The mean value of PASI after 20 doses of ultraviolet radiation was 6.17 (±3.60) and was significantly lower in comparison to mean PASI value before therapy (21.38 ± 9.84). On average, all patients improved their clinical condition, as measured by the PASI index, by 72% (43 patients had a PASI of 50; 22 patients had a PASI of 75; 3 patients had a PASI of 90). Moreover, the improvement was achieved in the range of BSA after 20 irradiations (13.90 ± 7.08 vs. 33.46 ± 12.71; *p* < 0.05) and the DLQI index, which decreased on average 1.60 ± 0.66 vs. 3.32 ± 0.79; *p* < 0.05.

The median values of TNF alpha, IL-17 and IL-23 in patients with psoriasis before phototherapy was significantly higher compared to the control group (10.45 pg/mL vs. 6.33 pg/mL, *p* = 0.003; 30.49 pg/mL vs. 14.65 pg/mL, *p* < 0.0001; 94.12 pg/mL vs. 64.41 pg/mL, *p* < 0.0001). The baseline levels of vitamin D amongst all the patients were 22.13 ± 12.52 ng/mL, whilst in the control group they were 19.33 ± 7.78 ng/mL. After the nb-UVB phototherapy, the average level was 35.03 ± 14.57 ng/mL for the patients. No statistical difference was observed in vitamin D level in patients with psoriasis compared to the control group (*p* > 0.05). There were no statistical differences in the parameters described above by gender in the individual groups (*p* > 0.05 for all comparisons). Statistical analysis shows that the PASI 75 response was dependent on changes in the concentrations of the analyzed cytokines (*p* > 0.05 for all comparisons).

### 3.2. Genotype Frequencies

The distribution of the analyzed *VDR* gene polymorphisms in patients with psoriasis and controls were in correspondence with the Hardy-Weinberg equilibrium (*p* > 0.05 in all cases), showing that the analyzed groups were selected correctly. When analyzing the distribution of genotypes of individual polymorphisms in this gene, no differences were found between the control group and patients, except for the GG genotype in the *TaaI*/Cdx-2 polymorphism which was significantly more frequent in patients with psoriasis (*p* < 0.001; OR 9.75). Other genotypes such as *BsmI* AA, *FokI* TT and CT increased the risk of psoriasis, although the relative risks for these associations were not so high (*p* = 0.03, *p* = 0.04, *p* = 0.03). Table 2 presents the distribution of *ApaI*, *FokI*, *TaqI*, *BsmI* and *TaaI*/Cdx-2 VDR genotypes in the patients with psoriasis and controls.

### 3.3. Association of VDR SNPs with Selected Clinical Features

When analyzing genotypes in individual polymorphisms for the VDR gene in terms of the therapeutic response to phototherapy, improvement of PASI 50, PASI 75 or PASI 90 did not show any relationship except with the *TaaI*/Cdx-2 polymorphism. We found a significantly higher frequency of rs11568820 AA (*TaaI*/Cdx-2) amongst the group with a negative response (*p* = 0.04, odds ratio 7.31, 95% confidence interval 1.19–44.97). The common rs11568820 AA (*TaaI*/Cdx-2) variant might have a significant effect on the response to therapies amongst patients with psoriasis (Table 3).

TNF alpha concentration in patients with psoriasis decreases significantly after UVB irradiation (10.45 pg/mL vs. 3.31 pg/mL; *p* < 0.01). The decrease in TNF alpha concentration was not dependent on polymorphisms in the *VDR* gene, except for the presence of the *TaaI*/Cdx-2 AA genotype and *TaaI*/Cdx-2 TT genotype of the *VDR* polymorphism, the presence of which was not associated with a statistically significant decrease in TNF alpha concentration (Table 4).

Irradiation with a narrowband UVB caused a decrease in the concentration of IL-17. The decrease in IL-17 concentration after phototherapy was statistically significant for patients with the CT genotype in the *FokI* polymorphism (*p* = 0.03), for the AA genotype in the *BmsI* polymorphism (*p* = 0.03) and in the GG polymorphism in *TaaI*/Cdx-2 (*p* = 0.03). In other genotypes of determined polymorphisms, no relationship was observed in the reduction of IL-17 after irradiation compared to other genotypes in the analyzed polymorphisms (Table 5).

NB-UVB irradiation may have impacted the IL-23 concentration. Moreover, the decrease in IL-23 concentration after phototherapy was statistically significant for patients with the TT genotype in the *FokI* polymorphism (*p* = 0.00001), for the CC genotype in the polymorphism *TaqI* (*p* = 0.003) and in the GG polymorphism in *TaaI*/Cdx-2 (*p* = 0.01). In other genotypes of the determined polymorphisms, no relationship was observed in the reduction of IL-23 after irradiation compared to other genotypes in the analyzed polymorphisms (Table 6).

Irradiation with NB-UVB in all patients caused a significant increase in vitamin D concentration, regardless of genotypes, in all analyzed VDR polymorphisms.

## 4. Discussion

The present study provides evidence of an association between *VDR* polymorphisms, cytokines, vitamin D levels and NB-UVB phototherapy for the first time. In our study, we analyzed an association between one of the *VDR* gene polymorphisms, level of TNF, IL-17, IL-23, vitamin D and psoriasis in the Polish population. Moreover, this was the first time the influence of *VDR* gene polymorphisms on effectiveness of phototherapy was explored. This data suggests that *VDR* polymorphisms may be involved in the etiopathogenesis of psoriasis and response to UVB therapy. The genotype distributions of all the analyzed polymorphisms of *VDR* are in the Hardy-Weinberg equilibrium.

*VDR* gene polymorphisms have been implicated in several autoimmune disorders, including systemic lupus erythematosus, rheumatoid arthritis and psoriasis [31,32,33,34].

Few studies have found a strong genetic background for psoriasis. We found that the frequency of the *TaaI*/Cdx-2 GG genotype was significantly higher in psoriasis patients than in controls. Other genotypes, such as *Bsml* AA, *Fokl* TT and CT, increased the risk of psoriasis, although the relative risks for these associations were not so high. Similarly, Zhu et al. [35] observed that *BsmI* polymorphism of the *VDR* gene is associated with psoriasis. In contrast, Xing Zhou et al. [36] and Rucevic et al. [34] did not find any significant differences between patients with psoriasis and controls in the genotype frequency of *BsmI*, *FokI*, and *TaaI*/Cdx-2 polymorphisms in Asian and Caucasian populations, respectively. There may be several reasons for this contradiction, probably due to different ethnic groups’ association with environmental factors, such as environmental exposure to UV radiation, or clinical heterogeneity of the study groups.

It is well known that biologically active 1,25-dihydroxyvitamin D_3_ regulates the growth of epidermal cells by inhibition of its proliferation and induces terminal differentiation of keratinocytes. Moreover, it activates anti-inflammatory and immunosuppressive pathways. Physiological response of keratinocytes and clinical response to treatment with vitamin D is correlated with the VDR mRNA expression, which may be influenced by the polymorphisms of the *VDR*. Polymorphism *BsmI* is located in intron 8 in the 3′ untranslated region (UTR), which may alter mRNA levels and the efficiency of protein translation by regulating gene transcription to change the expression and function of VDR. *FokI* is located in the 5′ end of the *VDR* gene and alters the start codon (ATG, Met1Thr) and leads to forming a protein with a different size—a shorter (424 amino acids) VDR protein—instead of a long (427 amino acids) VDR protein.

The 424-amino-acid VDR variant is more active than the 427-amino-acid variant in terms of its transactivation capacity as a transcription factor. Thus, the binding of vitamin D to VDR might be decreased and change the activation of the vitamin D/VDR signaling pathway. In that way, it inhibits the functions of VDR signaling pathway, such as anti-inflammatory effects, and leads to the overacting of the immune system. The *TaaI*/Cdx-2 polymorphism is located in the promoter region and plays a role in transcription of the *VDR* gene. Irradiation with UVB in all patients caused a significant increase in vitamin D concentration, regardless of genotypes, in all analyzed *VDR* polymorphisms. In patients with psoriasis, resistance to treatment with vitamin D was observed, which may be due to *VDR* gene polymorphisms. Our analysis showed an association between the *VDR TaaI*/Cdx-2 AA homozygous with a negative response to phototherapy, probably due to altered transcription of *VDR* gene but with normal production of vitamin D after UVB irradiation. Caudal-type homeobox protein 2 (Cdx-2) is a transcription factor (TF) with a polymorphic binding site (*TaaI*/Cdx-2) in the *VDR*. The molecular mechanism underlying the Cdx-2 association with conditions like psoriasis, which depends on VDR expression and vitamin D absorption, is believed to be due to higher affinity of Cdx-2 for the G allele compared to the A allele. *TaaI*/Cdx-2 with the AA genotype shows significantly lower VDR activation than AG and GG genotypes. Thus, the *TaaI*/Cdx-2 polymorphism might be a crucial functional polymorphism in the transcription of the *VDR* gene [17].

The rs7975232 *ApaI* variant of *VDR* may be associated with the development of inflammatory disease, such as oral lichen planus [37] and psoriasis [31,36] in the Asian population. Conversely, our study showed no association of *ApaI* variants with psoriasis in the Caucasian population.

In the case of psoriasis, as in most diseases with a complex etiology, the response to treatment is individual and often difficult to predict. This study was conducted to investigate whether *VDR* polymorphisms could be susceptibility markers for psoriasis. Therefore, screening of genetic markers will allow for the selection of the best treatment option for patients with psoriasis in the future, as well as decrease the treatment costs [5].

Interleukin-17 is a pro-inflammatory cytokine produced by the T helper 17 (Th17) cells and plays an important role in the development and progression of inflammatory and autoimmune diseases [38]. Mechanisms underlying the complex pathogenesis of psoriasis have not been fully understood, but there is increasing evidence that the IL-17/IL-23 axis plays a crucial role in the inflammatory response in psoriasis [39,40]. The vitamin D_3_ analogue calcipotriol used in treating psoriasis inhibited IL-23/IL-17 axis and neutrophil infiltration in psoriatic skin through the vitamin D receptor (VDR) in keratinocytes [41].

IL-23 alone promotes epidermal hyperplasia and activates the keratinocyte proliferation [42] or acts simultaneously with IL-17, enhancing dermal acanthosis, neutrophil recruitment and infiltration of IL-17-secreting cells into the psoriatic skin [6]. The median values of TNF alpha, IL-17 and IL-23 in patients with psoriasis before phototherapy was significantly higher compared to the control group. The PASI 75 response was dependent on changes in the concentrations of the analyzed cytokines. TNF alpha concentration in patients with psoriasis decreases after UVB irradiation, independent of polymorphisms in the *VDR* gene, except for the presence of the *TaaI*/Cdx-2 AA genotype and *TaqI* TT genotype. IL-17 and IL-23 are associated with inflammatory and dendritic cells such as Langerhans cells in skin. Irradiation with a narrowband UVB caused a decrease in the concentration of IL-17 and IL-23. The decrease in IL-17 concentration after phototherapy was observed for patients with the CT genotype in the *FokI* polymorphism and IL-23 for TT genotype. Decrease of IL-17 was observed for the AA genotype in the *BmsI* polymorphism and IL-23 for the CC genotype in the *TaqI* polymorphism. For the GG polymorphism in *TaaI*/Cdx-2, simultaneous decrease of the IL-17 and IL-23 level was observed.

A few limitations of the present study require consideration. First, the number of samples tested was relatively small and thus further studies in a larger population are required. We observed a weak association between genotypes *Bsml* and *FokI* and psoriasis, and lack of association with *ApaI*. Therefore, it is important to mention that the absence of a significant association of *VDR ApaI* polymorphism with psoriasis in the present study may be explained by the number of persons analyzed, which is insufficient for the investigation of rare alleles in the case of comparing Caucasians to Asians. Second, we did not evaluate interactions between vitamin D intake and environmental ultraviolet radiation exposure. Third, we did not evaluate associations with other relevant gene polymorphisms, IL-7, IL-23 or GC, and the vitamin D binding protein.

Strengths of this study include the collection of clinically characterized cohorts and results that were confirmed in two independent populations. Additionally, our study provides data for comparison with other studies to determine the possible value of *VDR* polymorphisms to predict predisposition to psoriasis in different ethnic groups.

## 5. Conclusions

Summarizing, we examined the association between *VDR* gene polymorphisms and psoriasis in the Polish population. This is the first report of a relationship between *VDR* polymorphisms and response to UVB treatment of psoriasis. The *TaaI*/Cdx-2 AA variant might have a significant effect on the response to phototherapy amongst patients with psoriasis. On the other hand, the *TaaI*/Cdx-2 GG variant might have an association with regulation of inflammatory response via decrease of the IL-17 and IL-23 level after UVB phototherapy in the Polish population. Moreover, *VDR* polymorphisms could be susceptibility markers for psoriasis and effectiveness of UVB therapy.

Further analysis of *VDR* gene polymorphisms may help to obtain more information on their association with development of psoriasis and different responses to phototherapy.

## Figures and Tables

**Table 1:** PCR-RFLP of loci within the *VDR* (vitamin D receptor) gene.

Polymorphism	Alleles	PCR Primer	Annealing Temperature	PCR Product (BP)	Restriction Enzyme	RFLP Products (BP)
rs2228570 (*FokI*)	162 T/C (Met1Thr, exon 1)	F: 5′-CACCCTGGAAGTAAAACA-3′ R: 5′-ACCTGAAGAAGCCTTTGC-3′	56 °C	486	FokI	486 CC 344/142 TT 486/344/142 TC
rs7975232 (*ApaI*)	64978G/T (intron 8 variant)	F: 5′- GCAAAGATAGCAGAGCAGAGTTCC –3′ R: 5′- AGGTTGGACAGGAGAGAGAATGG -3′	56 °C	781	ApaI	781 TT 469/312 GG 781/469/312 GT
rs1544410 (*BsmI*)	63980G/A (intron 8 variant)	F: 5′- GGGGAGTATGAAGGACAAAGAC–3′ R: 5′-TTCTCACCTCTAACCAGCGG-3′	56 °C	429	HinP1I	429 AA 282/147 GG 429/282/147 GA
rs731236 (*TaqI*)	1216 C/T (Ile352Ile, exon 9)	F: 5′- CAGAGCATGGACAGGGAGCAAG-3′ R: 5′-GCAACTCCTCATGGCTGAGGTCTC-3′	56 °C	740	TaqI	740 TT 495/245 CC 740/495/245 CT
rs11568820 (*TaaI*/Cdx-2)	G/A (promoter region)	TaqMan^®^ SNP Genotyping assay C___2880808_10				

**Table 2:** Distribution of *ApaI*, *FokI*, *TaqI*, *BsmI* and *TaaI*/Cdx-2 genotypes in the *VDR* gene amongst patients with psoriasis and controls.

		Controls		Patients with Psoriasis		*p* Value *
*ApaI*		N	(%)	N	(%)	
	GG	11	(22)	11	(22)	
	GT	17	(34)	20	(40)	
	TT	22	(44)	19	(38)	
*FokI*						
	CC	31	(62)	12	(24)	
	CT	11	(22)	21	(42)	0.03
	TT	8	(16)	17	(34)	0.04
*TaqI*						
	TT	17	(34)	15	(30)	
	TC	22	(44)	20	(40)	
	CC	11	(22)	15	(30)	
*BsmI*						
	AA	6	(12)	17	(34)	0.03
	AG	18	(36)	20	(40)	
	GG	26	(52)	13	(26)	
*TaaI*/Cdx-2						
	AA	23	(46)	7	(14)	
	AG	22	(44)	17	(34)	
	GG	5	(10)	26	(52)	<0.001

* *p* < 0.05 Bonferroni corrections.

**Table 3:** PASI and BSA response for various polymorphism of *VDR* gene.

		PASI 50 Yes		PASI 50 No		PASI 75 Yes		PASI 75 No		PASI 90 Yes		PASI 90 No		BSA Response over Median	
*Apal*		N	(%)	N	(%)	N	(%)	N	(%)	N	(%)	N	(%)	N (yes)	(%)
	GG	8	(73)	3	(27)	3	(27)	8	(73)	0	(0)	11	(100)	5	(45)
	GT	17	(85)	3	(15)	9	(45)	11	(55)	2	(10)	18	(90)	8	(40)
	TT	18	(95)	1	(5)	10	(53)	9	(47)	1	(5)	18	(95)	11	(58)
*Fokl*															
	CC	9	(75)	3	(25)	5	(42)	7	(58)	1	(8)	11	(92)	5	(42)
	CT	19	(90)	2	(10)	8	(38)	13	(62)	1	(5)	20	(95)	10	(48)
	TT	15	(88)	2	(12)	9	(53)	8	(47)	1	(6)	16	(94)	9	(53)
*Taql*															
	TT	14	(93)	1	(7)	6	(40)	9	(60)	2	(13)	13	(87)	7	(47)
	TC	18	(90)	2	(10)	11	(55)	9	(45)	1	(5)	19	(95)	11	(55)
	CC	11	(73)	4	(27)	5	(33)	10	(67)	0	(0)	15	(100)	6	(40)
*Bsml*															
	AA	15	(88)	2	(12)	10	(59)	7	(41)	1	(6)	16	(94)	8	(47)
	AG	16	(80)	4	(20)	7	(35)	13	(65)	2	(10)	18	(90)	8	(40)
	GG	12	(92)	1	(8)	5	(38)	8	(62)	0	(0)	13	(100)	8	(62)
*Taal*/Cdx-2															
	AA	4	(57)	3	(43)	2	(29)	5	(71)	0	(0)	7	(100)	1	(14)
	AG	15	(88)	2	(12)	6	(35)	11	(65)	0	(0)	17	(100)	7	(41)
	GG	24	(92)	2	(8)	14	(54)	12	(46)	3	(12)	23	(88)	16	(62)

**Table 4:** The comparison of TNF alpha before and after UV irradiation for various polymorphisms of the *VDR* gene.

		Before UV (Mean)	After UV (Mean)	*p*
*FokI*	TT	15.77	2.96	0.0130 *
	CT	10.03	2.75	0.0021 *
	CC	8.87	2.12	0.0404 *
*ApaI*	TT	11.43	3.58	0.0475 *
	TG	10.73	2.67	0.0006 *
	GG	11.14	2.64	0.0203 *
*TaqI*	TT	12.75	3.34	0.0742
	TC	10.96	2.24	0.0001 *
	CC	9.14	3.68	0.0013 *
*BsmI*	AA	16.09	2.89	0.0178 *
	GA	9.55	3.22	<0.0001 *
	GG	8.99	2.83	0.0214 *
*TaaI*/Cdx-2	GG	12.94	2.98	0.0066 *
	AG	9.09	2.59	<0.0001 *
	AA	11.51	4.11	0.1720

* statistical significance.

**Table 5:** The comparison of IL-17 before and after UV irradiation for various polymorphisms of the *VDR* gene.

		Before UV (Mean)	After UV (Mean)	*p*
*FokI*	TT	28.33	24.11	0.1177
	CT	28.32	22.89	0.0376 *
	CC	20.31	22.81	0.4713
*ApaI*	TT	23.52	23.41	0.9674
	TG	25.63	23.85	0.5515
	GG	27.57	22.04	0.1329
*TaqI*	TT	22.55	22.53	0.9958
	TC	25.26	23.72	0.5784
	CC	28.43	23.46	0.0726
*BsmI*	AA	28.48	22.21	0.0365 *
	GA	25.68	23.41	0.3910
	GG	22.06	24.49	0.4772
*TaaI*/Cdx-2	GG	28.76	23.79	0.0076 *
	AG	24.99	22.37	0.4618
	AA	19.82	23.62	0.3281

* statistical significance.

**Table 6:** The comparison of IL-23 before and after UV irradiation for various polymorphisms of the *VDR* gene.

		Before UV (Mean)	After UV (Mean)	*p*
*Fokl*	TT	95.35	75.53	<0.0001 *
	CT	94.24	88.24	0.3329
	CC	86.95	75.51	0.2028
*Apal*	TT	83.50	81.85	0.7814
	TG	88.40	85.30	0.6725
	GG	92.15	83.58	0.2697
*Taql*	TT	82.03	89.32	0.3626
	TC	89.18	83.31	0.3796
	CC	90.70	78.31	0.0320 *
*Bsml*	AA	88.16	79.23	0.1717
	GA	91.97	83.87	0.1134
	GG	81.25	88.94	0.4187
*TaaI*/Cdx-2	GG	96.16	86.13	0.0114 *
	AG	88.18	81.05	0.2374
	AA	71.55	80.46	0.5445

* statistical significance.

## Data Availability

The datasets generated during and analyzed during the current study are available from the corresponding author on reasonable request.
